# P-1110. The Impact of Xeruborbactam on *in vitro* Activity of Cefiderocol against a Panel of Enterobacterales Enriched with Isolates with Reduced Cefiderocol Susceptibility

**DOI:** 10.1093/ofid/ofae631.1298

**Published:** 2025-01-29

**Authors:** Takafumi Hara, Naoki Ishibashi, Dai Miyagawa, Motoyasu Onishi, Olga Lomovskaya, Yoshinori Yamano

**Affiliations:** Shionogi & Co., Ltd., Toyonaka, Osaka, Japan; Pharmaceutical Research Division, Osaka, Osaka, Japan; Shionogi & Co., Ltd., Toyonaka, Osaka, Japan; Shionogi & Co., Ltd., Toyonaka, Osaka, Japan; Qpex Biopharma Inc., San Diego, California; Shionogi & Co., Ltd., Toyonaka, Osaka, Japan

## Abstract

**Background:**

Cefiderocol (CFDC) is a siderophore cephalosporin with potent activity against resistant gram-negative bacteria. According to recent SIDERO-WT surveillance studies that involved testing of > 30,000 Enterobacterales (ENT) isolates collected in North America and Europe, 99.8% of ENT were susceptible (S) to CFDC (by CLSI breakpoint) (MIC_90_=1 µg/mL). For carbapenem-resistant Enterobacterales (CRE), S to CFDC was as high as 96.5% (MIC_90_=4 µg/mL). In ENT, resistance to CFDC is multifactorial and seems to require production of either a serine (SBL) or metallo (MBL) carbapenemase. Xeruborbactam (XER) is a novel beta-lactamase inhibitor that inhibits multiple SBL and MBL enzymes. In this study, we evaluated the impact of XER on activity of CFDC against a challenge panel of β-lactamase-producing ENT (BL-ENT) enriched in CFDC-resistant isolates.

MIC50/90 of cefiderocol (CFDC) alone and with xeruborbactam (XER) against Enterobacterales

**Figure:**
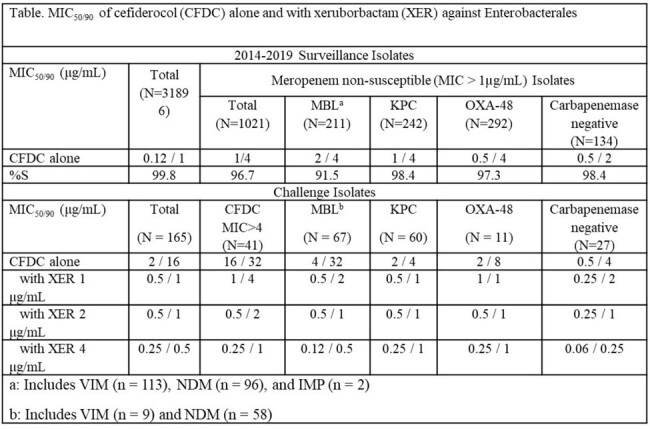

**Methods:**

A challenge panel of 165 BL-ENT isolates that included 139 CRE was used for this study. 79 (56.8%) and 41 (24.8%) of isolates had CFDC MIC values > 1 µg/mL and > 4 µg/mL (non-S by CLSI/FDA breakpoint), respectively. The β-lactamase profile for each isolate was determined by PCR or whole genome sequencing. MICs were determined for CFDC alone and in combination with 1, 2 or 4 μg/mL of XER according to CLSI guidelines for CFDC using iron-depleted CAMHB.

**Results:**

MIC_50_/MIC_90_ of CDFC alone and XER alone against 165 ENT were 2/16 and 16/ > 16 μg/mL, respectively. Dose-dependent shifts of CFDC MICs to the more susceptible range in the presence of increasing concentrations of XER were observed. XER at ≥ 1 μg/mL reduced the MIC_90_ of CFDC to ≤ 1 μg/mL, which is below the MIC_90_ for CFDC alone for all ENT in surveillance studies. XER at ≥ 2 μg/mL reduced CFDC MIC_90_ of MBL producers to ≤ 1 μg/mL For isolates with CFDC MIC > 4 μg/mL, 4 μg/mL of XER reduced the CFDC MIC_90_ from 32 to 1 μg/mL; the MIC_50_ for this subset of isolates were reduced from 16 to 0.25 µg/mL, the same MIC_50_ that were observed for all surveillance isolates.

**Conclusion:**

XER significantly improved the activity of CFDC against rarely encountered isolates of Enterobacterales with elevated CFDC MICs. The potency of CFDC/XER against these isolates was as high as potency of CFDC alone against CFDC susceptible strains.

**Disclosures:**

**Takafumi Hara, MSc**, SHIONOGI & CO., LTD.: Employee **Naoki Ishibashi, MD**, SHIONOGI & CO., LTD.: Employee **Dai Miyagawa, n/a**, SHIONOGI & CO., LTD.: Employee **Motoyasu Onishi, PhD**, Shionogi & Co., Ltd.: Employee **Olga Lomovskaya, PhD**, Qpex Biopharma Inc.: Board Member|SHIONOGI & CO., LTD.: Employee **Yoshinori Yamano, PhD**, Shionogi & Co., Ltd.: Employee

